# Augmenting Anti-Cancer Natural Products with a Small Molecule Adjuvant

**DOI:** 10.3390/md13010065

**Published:** 2014-12-26

**Authors:** Paul G. Wahome, Kevin R. Beauchesne, Anna C. Pedone, John Cavanagh, Christian Melander, Paul Zimba, Peter D. R. Moeller

**Affiliations:** 1 Biosortia Pharmaceuticals, 565 Metro Place South, Suite 300, Dublin, OH 43017, USA; E-Mails: pwahome@biosortia.com (P.G.W.); kbeauchesne@biosortia.com (K.R.B.); apedone@biosortia.com (A.C.P.); 2 Toxin/Natural Products Chemistry, National Ocean Service/NOAA, Hollings Marine Lab, 331 Fort Johnson Road, Charleston, SC 29412, USA; 3 Department of Molecular & Structural Biochemistry, North Carolina State University, Campus Box 7622, 128 Polk Hall, Raleigh, NC 27695, USA; E-Mail: john_cavanagh@ncsu.edu; 4 Department Chemistry, North Carolina State University, 2620 Yarbrough Drive, Box 8204, Raleigh, North Carolina 27695, USA; E-Mail: christian_melander@ncsu.edu; 5 Department of Life Sciences, Texas A&M Corpus Christi, 6300 Ocean Drive, Corpus Christi, TX 78412, USA; E-Mail: paul.zimba@tamucc.edu

**Keywords:** anti-cancer agents, cytotoxicity, natural products, toxins, adjuvant, small molecule

## Abstract

Aquatic microbes produce diverse secondary metabolites with interesting biological activities. Cytotoxic metabolites have the potential to become lead compounds or drugs for cancer treatment. Many cytotoxic compounds, however, show undesirable toxicity at higher concentrations. Such undesirable activity may be reduced or eliminated by using lower doses of the cytotoxic compound in combination with another compound that modulates its activity. Here, we have examined the cytotoxicity of four microbial metabolites [ethyl *N*-(2-phenethyl) carbamate (NP-1), Euglenophycin, Anabaenopeptin, and Glycolipid 652] using three *in vitro* cell lines [human breast cancer cells (MCF-7), mouse neuroblastoma cells (N2a), and rat pituitary epithelial cells (GH4C1)]. The compounds showed variable cytotoxicity, with Euglenophycin displaying specificity for N2a cells. We have also examined the modulatory power of NP-1 on the cytotoxicity of the other three compounds and found that at a permissible concentration (125 µg/mL), NP-1 sensitized N2a and MCF-7 cells to Euglenophycin and Glycolipid 652 induced cytotoxicity.

## 1. Introduction

Euglenophycin [1], Anabaenopeptin B [2,3] and Glycolipid 652 [4] are natural products isolated from aquatic microorganisms. All are under evaluation for use as anti-cancer agents. Euglenophycin, a toxin isolated from *Euglena sanguinea* [1], has exhibited cytotoxic activities [5]. Anabaenopeptins are known protein phosphatase inhibitors [2], while a novel glycolipid discovered in our lab has shown strong selective cytotoxicity against N2a and MCF-7 cells [4]. In many cases, combinations of these compounds, as we have found with Euglenophycin and the glycolipid in this present research, increase cytotoxic responses, which may result in undesirable toxicity. It was our aim to assess the use of these compounds as adjuvants to attenuate toxicity, thereby reducing the amount of toxin while retaining the same effectiveness or desired activity. Compounds such as NP-1 and their derivatives are effective biofilm inhibitors and have been proven to be more effective when combined with antibiotics [6]. These findings prompted us to examine the potential adjuvant effect of NP-1 on cytotoxic compounds.

In this study, we report the use of NP-1 in magnifying the cytotoxicity of compounds derived from aquatic microbes. This increased activity has implications in areas such as cancer treatment where a desired outcome of drug design is reducing toxic side effects. This is particularly important in pediatric cancer therapy where such side effects may lead to complications later in life, including impaired growth and development, endocrine, gastrointestinal, and neurocognitive dysfunction, cardiopulmonary compromise, renal impairment, and subsequent malignancies [7].

## 2. Results

### 2.1. Test Compounds

**Figure 1:**
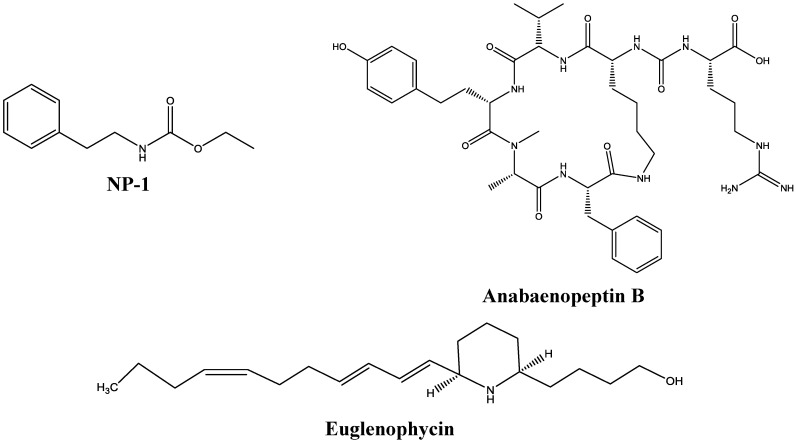
Chemical structures of select compounds used in this study.

We previously isolated natural compounds that had promise as good cancer treatment candidates [1,4]. These compounds include NP-1, Euglenophycin, Anabaenopeptin B, and Glycolipid 652 (Figure 1, [4]). The purification and structural characterization data for Glycolipid 652 have not been reported but can be provided upon request. Anabaenopeptin B is a cyclic hexapeptide that was originally isolated from a fresh water cyanobacterium, *Anabaena flos-aquae*, while Glycolipid 652 was isolated by Biosortia Pharmaceuticals from a consortium of fresh water microbes [4]. On the other hand, NP-1 is a metabolite from the marine bacterium *Cytophaga* sp. [6,8].

In this study, we examined the toxicity of these compounds on three different cell lines individually and then evaluated the modulation of three of the compounds (Euglenophycin, Anabaenopeptin B, and Glycolipid 652) by NP-1. The purity of the compounds was established to be ≥95% by multiple LC/MS and NMR analyses.

### 2.2. Neuro-2a Cells Are Sensitive to Euglenophycin and Glycolipid 652 Induced Toxicity

**Figure 2:**
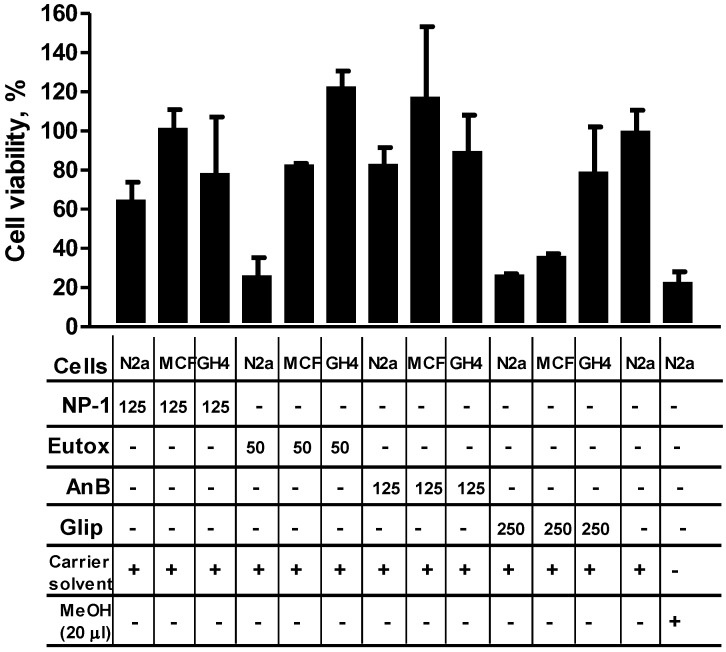
The effect of treating Neuro-2a (N2a), Michigan Cancer Foundation 7 (MCF-7), or epithelial cells (GH4) with the test compounds (NP-1, Euglenophycin (Eutox), Anabaenopeptin B (AnB), and Glycolipid 652 (Glip)). Controls are shown as the effect of the carrier solvent (negative control; 1.9% MeOH final concentration) or 20 µL MeOH (positive control; 66.67% final concentration). Cells treated with varying concentrations (µg/mL) of the test compounds were incubated at 37 °C for 24 h before their viability was determined as described in the Experimental Section. The results shown are for a representative experiment that was performed in duplicate. Error bars show percent correlation of variation (% CV) for replicate wells. The cytotoxicity data includes select test concentrations that produced measurable differences in activity. Symbols: + (plus sign), added to the test well; − (minus sign), not added to the test well.

**Figure 3:**
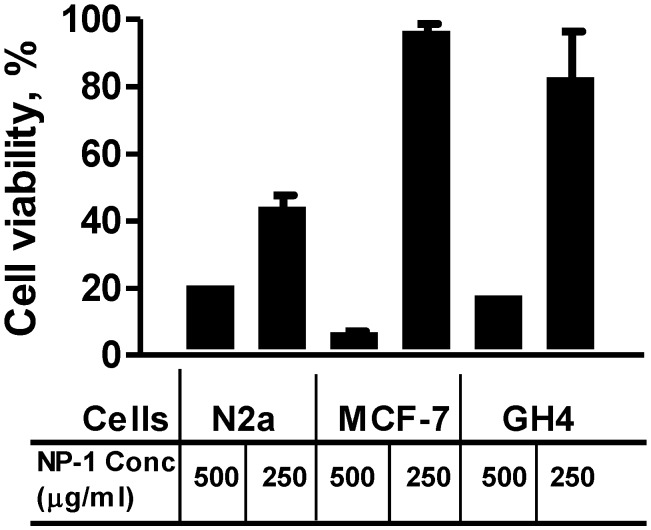
The effect of treating N2a, MCF-7, or GH4 cells with 500 or 250 µg/mL NP-1. Cells treated with 500 or 250 µg/mL NP-1 were incubated at 37 °C for 24 h before their viability was determined as described in the Experimental Section. The results shown are for a representative experiment that was done in duplicate. Error bars show percent correlation of variation (% CV) for replicate wells.

We began this study by examining the effect of varying concentrations of NP-1, Euglenophycin, Anabaenopeptin B, and Glycolipid 652 on the proliferation of Neuro-2a (N2a), MCF-7, and GH4C1 (GH4) cells using the MTT Cell Proliferation Assay (ATCC; Manassas, VA, USA). This assay measures cell proliferation rate, and metabolic events that lead to apoptosis and necrosis cause a reduction in cell viability. Neuronal cells (N2a), epithelial cells (GH4) and human breast cancer cells (MCF-7), three *in vitro* cell lines important in both cell biology and oncology, were used. The toxicity of the compounds was evaluated at concentrations ranging from 4 to 500 µg/mL (final concentration). The cells were separately seeded in 96-well microplates and incubated overnight (>16 h) at 37 °C (5% CO_2_ and 95% humidified air) before treatment with varying doses of the compounds. After 24 h incubation at 37 °C post treatment, the cell viability was determined using the MTT assay as previously described [9]. Prior to the addition of the MTT substrate, the treated cells were examined using a microscope to check for characteristic cell changes associated with cell death (*i.e.*, disruption of the monolayer, cell rounding, and lysing). NP-1 did not show dramatic cytotoxicity against N2a, MCF-7, or GH4 cells at concentrations 125 µg/mL or lower (Figure 2 and data not shown); however, potent cytotoxicity was noted in all three cell lines at the highest concentration (500 µg/mL) (Figure 3).

**Figure 4:**
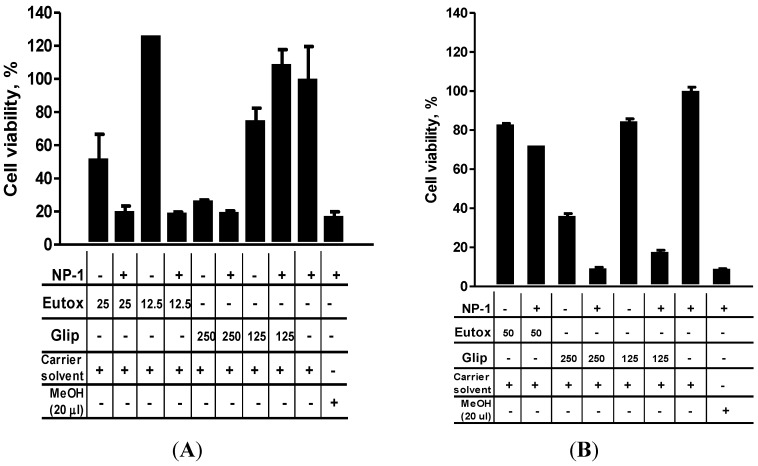
The effect of treating N2a (**A**) and MCF-7 (**B**) cells with Euglenophycin (Eutox) and Glycolipid 652 (Glip) in the presence and absence of 125 µg/mL NP-1. Controls are shown as the carrier solvent (negative control; 1.9% MeOH final concentration) or 20 µL MeOH (positive control; 66.67% final concentration) in the presence of 125 µg/mL NP-1. The treated cells were incubated at 37 °C for 24 h before their viability was determined as described in the Experimental Section. The results shown are for a representative experiment that was performed in duplicate. Error bars show percent correlation of variation (% CV) for replicate wells. The cytotoxicity data includes select test concentrations (µg/mL) that produced measurable differences in activity. Symbols: + (plus sign), added to the test well; − (minus sign), not added to the test well.

Euglenophycin showed a dose-dependent toxicity on N2a cells with an estimated 70% and 50% loss in cell viability at 50 and 25 µg/mL, respectively (Figure 2, Figure 3 and Figure 4A, and Supplementary Figure S1). In contrast, Euglenophycin showed only modest toxicity on MCF-7 cells and notable viability enhancement on GH4 cells at those concentrations (Figure 2 and Supplementary Figure S1). Anabaenopeptin B showed no significant toxicity on all three cell lines, even at 500 µg/mL and with prolonged incubation (7 days) at 37 °C (Figure 2 and data not shown). Glycolipid 652 showed no significant cytotoxicity on GH4 cells, but it caused dose-dependent toxicity on N2a cells with an estimated 75% loss in cell viability at 250 µg/mL and a 65% loss in cell viability for MCF-7 cells (Figure 2 and Supplementary Figure S2).

### 2.3. NP-1 Enhances Euglenophycin and Glycolipid 652 Induced Cytotoxicity

The results of the cytotoxicity assay (Section 2.2) showed that NP-1 is notably toxic to the *in vitro* cell lines only at concentrations greater than 250 µg/mL (Figure 3). These results together with those reported elsewhere [6] prompted us to evaluate whether NP-1 could enhance Euglenophycin or Glycolipid 652 induced cytotoxicity. Anabaenopeptin B was also included in this assay, although it had not shown significant toxicity on the cell lines (Section 2.2). N2a, MCF-7, and GH4 cells were separately seeded in 96-well plates and incubated overnight (>16 h) at 37 °C (5% CO_2_ and 95% humidified air) before being treated with 125 µg/mL (final concentration) of NP-1. This concentration of NP-1 had no significant effect on the viability of MCF-7 and demonstrated only modest toxicity on the N2a and GH4 cells (Figure 2). At the beginning of this study, we established that the cells must be preincubated with NP-1 for 20 h prior to treatment with the other test compounds for the cell viability enhancement to be noticeable. Loading NP-1 and a test compound at the same time or loading them at different times but within a short duration of time (e.g., 1 h) did not significantly alter the cytotoxicity of the test compound. Therefore, after a 20 h incubation at 37 °C, varying concentrations of Euglenophycin, Glycolipid 652 or Anabaenopeptin B were added to the cells. The cells were then incubated for an additional 24 h before their viability was determined using the previously described MTT assay [9]. Strikingly, the N2a cells pretreated with 125 µg/mL NP-1 showed increased sensitivity to Euglenophycin (Figure 4A). For instance, in the presence of NP-1 and 12.5 or 25 µg/mL Euglenophycin, N2a cells showed approximately 80% loss in cell viability. Similarly, treatment of N2a cells with NP-1 and 250 µg/mL Glycolipid 652 caused nearly an 80% loss in cell viability, although the presence of NP-1 had only a modest (<10%) effect on the cytotoxicity of Glycolipid 652. At a lower concentration of Glycolipid 652 (125 µg/mL), however, the presence of NP-1 caused enhanced cell proliferation (Figure 4A). In contrast to the sensitivity shown by N2a cells upon treatment with 25 µg/mL Euglenophycin and 125 µg/mL NP-1, MCF-7 cells were not affected. However, in the presence of NP-1 and 50 µg/mL Euglenophycin, we noted an approximate 10% reduction in the viability of MCF-7 cells (Figure 4B). In contrast, GH4 cells treated with varying concentrations of Euglenophycin showed enhanced viability, even at the highest concentration tested (500 µg/mL), and 125 µg/mL NP-1 did not alter the results obtained with Euglenophycin alone (data not shown). Anabaenopeptin B did not demonstrate cytotoxic effects on the three cell lines, even at the highest concentration (500 µg/mL) tested, and the presence of NP-1 did not change the results obtained with Anabaenopeptin B alone (Figure 2 and data not shown). Treatment of MCF-7 cells with 62.5 µg/mL Glycolipid 652 resulted in modest (~15%) loss of cell viability and only minimal change occurred in the presence of 125 µg/mL NP-1 (Supplementary Figure 2 and data not shown). Similarly, 125 µg/mL Glycolipid 652 caused a slight decrease (~15%) in viability of MCF-7 cells. However, in the presence of NP-1 and 125 µg/mL Glycolipid 652, there was an approximate 80% loss in cell viability. Moreover, the toxicity enhancement by NP-1 was evidenced by the dramatic loss (~90%) in the viability of MCF-7 cells at 250 µg/mL Glycolipid 652 (Figure 4B and Supplementary Figure S2). Overall, all cell lines showed potential selectivity and variability in response to compound exposure, but the toxicity enhancement by NP-1 was only noted in the N2a and MCF-7 cell lines.

## 3. Experimental Section

### 3.1. Materials

Neuro-2a (ATCC^®^ CCL-131™) and GH4C1 (ATCC^®^ CCL-82.2™) cells were obtained from the American Type Culture Collection (ATCC; Manassas, VA, USA). Michigan Cancer Foundation 7 (MCF-7) cells were from the National Cancer Institute (Bethesda, MD, USA). Calcium- and magnesium-free phosphate-buffered saline was obtained from MP Biomedicals (Solon, OH, USA). Trypsin (0.05%)-EDTA (0.53 mM) in Hanks’s balanced salt solution (HBSS) without sodium carbonate, calcium and magnesium was obtained from Mediatech, Inc. (Manassas, VA, USA). Cell culture flasks (T75 and T175 cm^2^ with canted/angled neck and 0.2 µM lid-fitted filter) and 96-well microplates (natural, flat bottom, sterile, and polystyrene tissue culture treated) were from Corning Inc. (Corning, NY, USA). NP-1 was obtained from Agile Sciences, Inc. (Raleigh, NC, USA). Euglenophycin was purified from axenic culture of *Euglena sanguinea* as described [1]. Anabaenopeptin B and Glycolipid 652 were provided by Biosortia Pharmaceuticals (Dublin, OH, USA). Methanol-d_4_ and dimethylsulfoxide-d_6_ were from Cambridge Isotope Laboratories, Inc. (Tewksbury, MA, USA). 3-(4,5-Dimethyl-2-thiazolyl)-2,5-diphenyl-2H-tetrazolium bromide (MTT), sodium dodecyl sulfate (SDS), and hydrochloric acid (HCl) were from Sigma Aldrich (St. Louis, MO, USA).

### 3.2. Test Compound Analyses

The identity, integrity and purity of the compounds used in this study were verified using Liquid Chromatography-Mass Spectrometry (LC-MS) and Proton Nuclear Magnetic Resonance (^1^H NMR) as described [1]. LC-MS was performed using a Waters 1525 system equipped with the 2767 sample manager and a ZQ mass spectrometer. ^1^H NMR was performed using a Bruker DMX 500 MHz spectrometer equipped with a 5 mm triple nucleus gradient probe, and data processing was performed using TopSpin 2.1 software. Compounds were dissolved in methanol-d_4_ or dimethylsulfoxide-d_6_ with deuterium serving as the lock nucleus.

### 3.3. Cell Lines and Cell Culture

N2a and MCF-7 cells were routinely grown and maintained in Eagle’s Minimum Essential Medium (EMEM) supplemented with 10% Fetal Bovine Serum (FBS), while GH4 cells were grown and maintained in Hams F10 medium supplemented with 15% horse serum and 2.5% FBS. All cells were grown in antibiotic-free media (to prevent interaction of the test compounds with the antibiotics, which may influence assay results) at 37 °C in a humidified atmosphere containing 5% CO_2_.

### 3.4. Cytotoxicity Assay

The N2a, GH4, and MCF-7 cells were each suspended in the appropriate growth medium and seeded (100 µL) in 96-well microplates at densities of 42,000, 50,000, and 42,000 cells/well, respectively. The cells were incubated at 37 °C overnight in a humidified atmosphere containing 5% CO_2_ to allow cell adherence to the bottom of the wells. A total of 4 µL of 2-fold serially diluted test compounds (13 to 0.102 mg/mL in 50% MeOH, which corresponded to 500 to 4 µg/mL final concentrations) was added to the cells in duplicate, while an equal volume of the carrier solvent (50% MeOH) was added to the negative control wells in duplicate. MeOH (20 µL of 100%) was added to the positive control wells. The cells were further incubated at 37 °C for 24 h before their viability was determined using the MTT procedures described by Schock *et al.* [9]. Briefly, the MTT (15 µL of a 5 mg/mL solution in phosphate buffered saline) was added to individual test wells and incubated at 37 °C for 4 h before SDS containing 0.1% HCl was added to solubilize the purple-colored product (formazan). After further incubation at 37 °C for 2 h, the optical density 570 nm (OD_570nm_) was measured for the test wells using a spectrophotometer (Molecular Devices, Sunnyvale, CA, USA). In this assay, the yellow MTT substrate is reduced to a purple colored formazan by mitochondrial dehydrogenases. Test wells with living and healthy cells show a strong purple color, while test wells with dead cells remain yellow. For our methodology, we continued visual examination of the plates for color change for a period of one week. Data were recorded and processed using SpectraMax^®^ Pro (Molecular Devices), Microsoft Excel 2010 (Redmond, WA, USA), and GraphPad Prism 5 (La Jolla, CA, USA) software. The cell viability was determined by dividing the average OD_570nm_ of replicate test wells with that of the cells treated with the carrier solvent and then multiplying this ratio by 100. The viability of cells treated with the carrier solvent was similar to that of cells not subjected to any treatment, whose viability was assumed to be 100%.

### 3.5. Cytotoxicity Enhancement by NP-1

The N2a, GH4, and MCF-7 cells were seeded in separate 96-well microplates and incubated overnight at 37 °C in a humidified atmosphere containing 5% CO_2_. The cells were then pretreated with 125 µg/mL (final concentration) NP-1 for 20 h before varying concentrations of Euglenophycin (100 to 0.8 µg/mL final concentrations), Anabaenopeptin B (500 to 4 µg/mL final concentrations) or Glycolipid 652 (500 to 4 µg/mL final concentrations) were added to the cells in duplicate. For the control wells, the cells were pretreated with NP-1 as described above before 4 µL of the carrier solvent, 50% MeOH (negative control), or 20 µL of 100% MeOH (positive control) was added in duplicate. The cells were further incubated for 24 h before their viability was determined using MTT, as described above and by Schock *et al.* [9].

## 4. Discussion and Conclusions

In the present study, we examined the cytotoxicity of 4 natural products (NP-1, Euglenophycin, Anabaenopeptin B, and Glycolipid 652) from aquatic microbes against three cancer cell lines including MCF-7, a human breast cancer cell line. We are currently evaluating these compounds for anti-cancer and anti-microbial activities. Earlier anti-microbial studies using NP-1 showed promising biofilm inhibition activities against various bacteria, including multidrug resistant *Staphylococcus aureus* (MRSA) and vancomycin-resistant *Enterococcus faecium* (VRE).

The results herein show that NP-1 and Glycolipid 652 did not cause significant cytotoxicity against MCF-1, N2a and GH4 cells at 125 µg/mL but were toxic at higher concentrations (250 and 500 µg/mL). Anabaenopeptin B was a relatively nontoxic to these cell lines at 125 µg/mL, but unlike NP-1 and Glycolipid 652, it was nontoxic at the higher concentrations. In contrast, Euglenophycin showed potent cytotoxicity against N2a cells, with an estimated 50% reduction in cell viability at a concentration of 25 µg/mL. Euglenophycin exhibited selectivity for N2a cells as it did not show significant cytotoxicity against the other two cell lines, even at the highest concentration tested (100 µg/mL). We also noted cell viability enhancement in more than one cell line when the cells were treated with the test compounds. We attribute this to cell proliferation enhancement and/or assay interference by the compounds.

Cytotoxic compounds have the potential to be lead compounds or drugs for cancer treatment. Many times, however, these compounds show undesirable toxicity at higher concentrations that result in their elimination from the drug development pipeline. This undesirable activity may be reduced or eliminated by using lower doses of the cytotoxic compound in combination with another compound(s) that modulates its activity. The results of our previous biofilm inhibitory studies [6] and the current cytotoxicity results advocate for the potential use of NP-1 (a relatively nontoxic compound at 125 µg/mL) to modulate the effects of a highly toxic compound, such as Euglenophycin, to reduce the negative cytotoxic effects from higher doses. To explore this potential outcome, we pretreated three cell lines with 125 µg/mL NP-1, and incubated the cells for 20 h before treatment with various doses of Euglenophycin, Anabaenopeptin, and Glycolipid 652. We noted increased sensitivity of N2a and MCF-7 cells to Euglenophycin- and Glycolipid-induced cytotoxicity. In developing the protocol for testing the modulatory effect of NP-1, we determined that the cell lines required overnight incubation with the compound prior to testing with the selected compounds. Immediate addition of the test compound with NP-1 to the cells resulted in identical cytotoxic activity observed with the test compound(s) alone. We interpret this result as NP-1 potentially affecting the cell membrane in some manner, particularly with respect to modulation of voltage-gated sodium channels (VGSC). This is supported by data showing that N2a and MCF-7 cells have elevated levels of VGSC, while GH4 cells have abundant levels of voltage-dependent calcium channels (VDCC) [10,11,12]. Our data for both the N2a and MCF-7 cell lines suggest an adjuvant type relationship between Euglenophycin or Glycolipid 652 and the activity of NP-1. Our continued work in this area includes optimizing dose responses to varying amounts of adjuvant and assessing other, more effective carbamate analogs with different cell lines. Though a great deal of work remains, the promise of more efficient human health treatments using NP-1 derived adjuvants to attenuate toxicity is very exciting.

## Supplementary Files

Supplementary File 1

## NOAA Disclaimer

This publication does not constitute an endorsement of any commercial product or intend to be an opinion beyond scientific or other results obtained by the National Oceanic and Atmospheric Administration (NOAA). No reference shall be made to NOAA, or this publication furnished by NOAA, to any advertising or sales promotion which would indicate or imply that NOAA recommends or endorses any proprietary product mentioned herein, or which has as its purpose an interest to cause the advertised product to be used or purchased because of this publication.
