# Supplementary material for: Augmenting Anti-Cancer Natural Products with a Small Molecule Adjuvant
Source: Mar Drugs. 2014 Dec 26;13(1):65–75. doi: 10.3390/md13010065 (PMC4306925; doi:10.3390/md13010065)
Supplement: Supplementary File 1 [file marinedrugs-13-00065-s001.pdf]

Supplementary Information

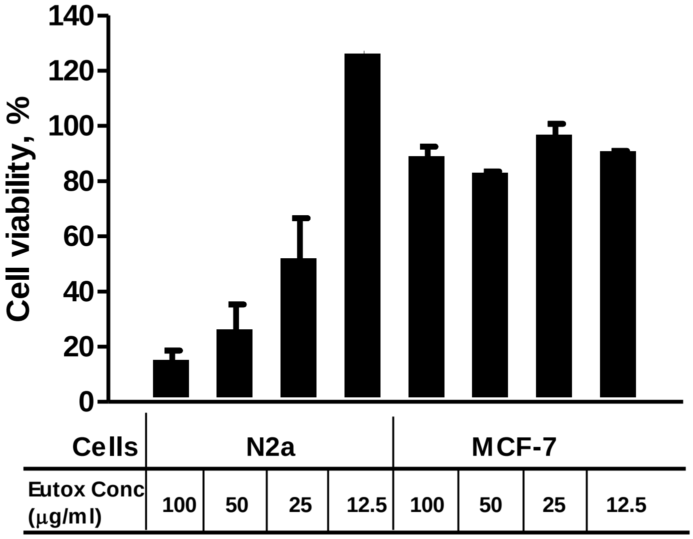

Figure S1. The effect of treating N2a or MCF-7 with varying concentrations of Euglenophycin (Eutox). Cells treated with 100, 50, 25 or 12.50 μg/mL Euglenophycin were incubated at 37 °C for 24 h before their viability was determined as described in the Experimental Section. The results shown are for a representative experiment that was performed in duplicate. Error bars show percent correlation of variation (% CV) for replicate wells.

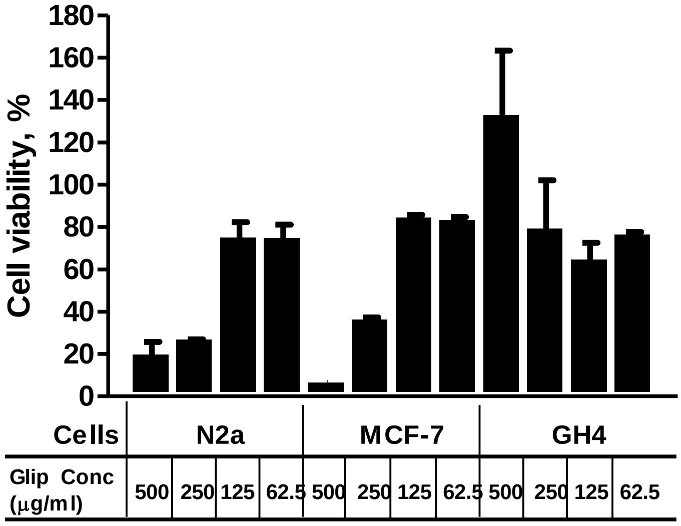

Figure S2. The effect of treating N2a, MCF-7, or GH4 cells with varying concentrations of Glycolipid 652 (Glip). Cells treated with 500, 250, 125, or 62.5 μg/mL Glycolipid 652 were incubated at 37 °C for 24 h before their viability was determined as described in the Experimental Section. The results shown are for a representative experiment that was performed in duplicate. Error bars show percent correlation of variation (% CV) for replicate wells.
